# Surface modification strategies of oral liposomes: functional design and barrier enhancement

**DOI:** 10.3389/fphar.2026.1801527

**Published:** 2026-06-05

**Authors:** Pengyu Jin, Siyuan Wu, Ying Wang, Yiran Ni, Qingxiao Ruan, Ziang Yuan, Haiping Yao, Junming Li

**Affiliations:** 1 The First College of Clinical Medical Sciences, China Three Gorges University, Yichang, Hubei, China; 2 Institute of Organ Fibrosis and Targeted Drug Delivery, China Three Gorges University, Yichang, Hubei, China; 3 Yichang Central People’s Hospital, Yichang, Hubei, China; 4 Hubei Key Laboratory of Ischemic Cardiovascular Disease, Yichang, Hubei, China

**Keywords:** bioavailability, liposomes, nanocarriers, oral drug delivery, surface modification, targeted delivery

## Abstract

Oral administration is the most prevalent and preferred clinical route due to its non-invasiveness, high patient compliance, and convenience. However, the oral delivery of many therapeutic drugs is hindered by low bioavailability, attributed to multiple gastrointestinal (GI) barriers including acid degradation, enzymatic hydrolysis, poor epithelial permeability, and first-pass metabolism. Liposomes have emerged as promising oral nanocarriers owing to their biocompatibility, versatile drug-loading capacity, and biomimetic membrane structure. Nevertheless, their poor physicochemical stability and inadequate cargo protection in the harsh GI environment limit clinical applications. This review summarizes the latest advances in surface modification strategies for liposomes to address these challenges. Synthetic polymer modifications (e.g., PEG, TPGS, pH-responsive Eudragit, and polydopamine) significantly boost the physicochemical stability of liposomes, prevent drug efflux, and improve mucus penetration. Natural biomacromolecule modifications (e.g., natural polysaccharides, proteins, peptides, and aptamers) effectively enhance mucoadhesion, cellular internalization, and active targeting capabilities. Meanwhile, small-molecule ligand modifications (e.g., folic acid, vitamin B12, and bile acids) actively promote intestinal transcytosis and targeted absorption by hijacking specific endogenous transporters. Notably, composite or multi-layer modification strategies (e.g., layer-by-layer assembly) achieve synergistic effects in effectively overcoming successive GI barriers. Furthermore, this review addresses the critical translational hurdles from bench to bedside, emphasizing that overcoming industrial scale-up bottlenecks (e.g., *via* microfluidic technologies) and conducting rigorous long-term biosafety evaluations are pivotal for the future clinical and commercial success of these advanced nanocarriers. Ultimately, these sophisticated surface engineering technologies remarkably enhance the physicochemical integrity, mucus penetration ability, and cellular uptake efficiency of liposomes, laying a solid foundation for translating efficient oral nanotherapeutics from bench to market.

## 1 Background

Oral administration is the most commonly used and preferred route in clinical practice, featuring non-invasiveness, high patient compliance, ease of use, and self-administration. Statistics show that over 60% of marketed drugs are delivered orally. Compared with injection and other routes, oral administration also offers advantages such as low cost and reduced risk of cross-infection (Alqahtani et al., 2021). However, despite these merits, oral delivery of biomacromolecular drugs (e.g., peptides, proteins, nucleic acids) and some small-molecule drugs is hindered by multiple gastrointestinal (GI) barriers. These barriers include chemical degradation by gastric acid and digestive enzymes, entrapment by the mucus layer, low intestinal epithelial permeability, hepatic first-pass metabolism, and drug metabolism by gut microbiota, resulting in extremely low oral bioavailability. For instance, even approved oral biologics (e.g., octreotide, semaglutide) exhibit bioavailability as low as 0.4%–1.0%, highlighting the limitations of traditional formulations in overcoming these complex physiological barriers.

To address these obstacles and improve oral absorption efficiency, various lipid-based nanocarriers have been developed, including liposomes, solid lipid nanoparticles, nanoemulsions, microemulsions, nanocapsules, and self-emulsifying drug delivery systems. These novel drug delivery systems have garnered extensive attention due to their low toxicity, excellent biocompatibility, scalability, and high drug-loading capacity. For instance, systems like solid lipid nanoparticles and self-emulsifying drug delivery systems can overcome gastrointestinal biochemical barriers by utilizing lipid matrices to solubilize and tightly encapsulate hydrophobic drugs, while simultaneously stimulating intestinal lymphatic transport to bypass hepatic first-pass metabolism. However, despite their success in enhancing the bioavailability of poorly water-soluble molecules, their rigid solid cores or high-surfactant compositions often limit their ability to protect and deliver fragile hydrophilic biologics, such as proteins and peptides (Liu et al., 2025). Among them, liposomes are regarded as one of the most promising oral nanocarriers owing to their unique bilayer structure and superior biocompatibility. They not only protect encapsulated drugs, prolong half-lives, and enable controlled release, but their biomimetic membrane structure also facilitates interactions with cell membranes. Additionally, they promote lymphatic transport to bypass hepatic first-pass metabolism, significantly improving bioavailability. Furthermore, diverse surface modification strategies are extensively employed to optimize the *in vivo* fate of oral liposomes. As a paradigmatic example of this modifiability, PEGylation (modification with polyethylene glycol) creates a highly hydrated steric barrier around the vesicles. This hydrophilic shield not only enhances mucus penetration within the GI tract but also significantly reduces rapid recognition and uptake by the reticuloendothelial system once absorbed. By endowing these nanocarriers with excellent stealth characteristics and non-immunogenic properties, PEGylation effectively mitigates premature immune clearance and prolongs their systemic circulation time, thereby establishing a crucial foundation for subsequent active targeting functionalizations (Suk et al., 2016; Janani et al., 2026). Driven by such unparalleled structural versatility, compared with other materials, liposomes excel in physicochemical stability, cargo protective capabilities, drug loading versatility, and safety, making them particularly suitable for the oral delivery of both fragile proteins/peptides and poorly soluble small-molecule drugs (Liu et al., 2025; Plaza-Oliver et al., 2021). These characteristics render liposomes an ideal nanoplatform for efficient oral drug delivery.

**FIGURE 1:**
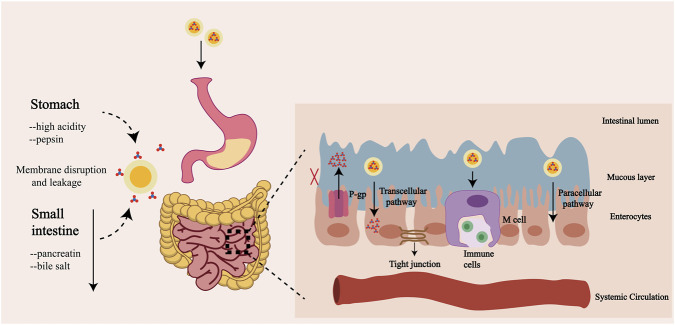
Schematic illustration of multiple physiological barriers limiting oral liposome absorption in the gastrointestinal tract.

Nevertheless, liposomes still face multiple challenges in the complex GI environment, mainly encompassing three aspects: destruction by physicochemical environments, obstruction by the mucus barrier, and low absorption efficiency of epithelial tissues. Specifically, the harsh physicochemical environment of the GI tract directly damages the liposome structure, leading to premature drug leakage. The strongly acidic environment (pH 1.2–3.0) and pepsin in the stomach can disrupt the liposome bilayer, while pancreatic enzymes (e.g., trypsin, phospholipase A2) and bile salts in the small intestine further accelerate phospholipid degradation. As biological surfactants, bile salts can insert into the liposome membrane and induce its decomposition into mixed micelles, causing drug release. Moreover, high osmotic pressure and shear forces from fluid flow in the GI tract also threaten liposome physicochemical stability. Secondly, the mucus layer covering the GI epithelium forms a dense three-dimensional network that effectively traps nanoparticles such as liposomes, restricting their diffusion to the epithelial surface. Meanwhile, the high turnover rate of the mucus layer continuously clears trapped particles, significantly shortening the contact time between liposomes and epithelial cells and further limiting absorption. Finally, even if liposomes cross the mucus layer to reach epithelial cells, their absorption still encounters numerous barriers. In the transcellular pathway, liposomes may enter cells *via* endocytosis but are prone to degradation by lysosomes. The paracellular pathway is severely restricted by tight junctions between epithelial cells, making effective transport difficult. Additionally, the low permeability of intestinal epithelial cells and the efflux of drugs by transporters such as P-glycoprotein (P-gp) result in drug excretion or metabolism before absorption. Moreover, absorptive cells such as M cells account for less than 1%, which cannot be relied upon as the main absorption pathway. These factors collectively lead to significantly reduced oral bioavailability of liposomes (Figure 1) (Daeihamed et al., 2017; Muhlberg et al., 2021).

Therefore, improving the physicochemical stability, cargo protective effects, and mucus penetration ability of liposomes in the GI environment while maintaining their biocompatibility has become a core issue in current oral liposome research. Liposome modification, as an effective strategy, has been extensively explored. Liposome modification strategies can be categorized into two types: bilayer composition modification and surface modification. These two strategies focus on overcoming multiple physiological and biochemical barriers during oral delivery. The core idea of bilayer composition modification is to fundamentally enhance the intrinsic physicochemical stability of liposomes in the harsh GI environment by altering the lipid components of the liposome membrane itself. The goal is to resist the destruction of the lipid bilayer by gastric acid, digestive enzymes, and bile salts, preventing premature drug leakage. Surface modification strategies do not change the internal lipid skeleton of liposomes but instead introduce one or more layers of functional materials on their surface to endow liposomes with new properties, mainly addressing issues such as mucus penetration, cellular uptake, and targeting (Muhlberg et al., 2021). Subsequent sections of this review will focus on surface modification strategies of liposomes, comprehensively elaborating on their mechanisms of action and latest research progress in breaking through oral delivery barriers.

## 2 Surface modification strategies of liposomes

**FIGURE 2:**
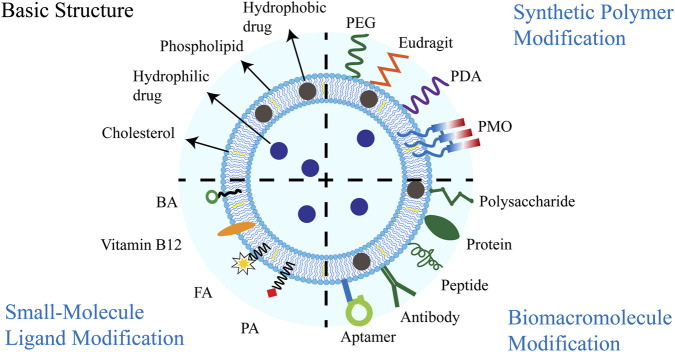
Schematic representation of diverse surface modification strategies for oral liposomes: synthetic polymer, biomacromolecule, and small-molecule ligand modifications.

As the foundational platform for these strategies, liposomes are self-assembling spherical vesicles primarily composed of phospholipids—which form the bilayer backbone—and cholesterol, which modulates membrane fluidity and enhances stability. Although their basal drug encapsulation efficiency and release behaviors can be dictated by formulating them into distinct sizes and lamellarities (such as small unilamellar, large unilamellar, and multilamellar vesicles), relying solely on this internal skeleton is often insufficient to fully overcome the complex oral delivery barriers (Durgadevi et al., 2026). Surface modification technology involves introducing functional materials (e.g., polymers, biomacromolecules, or small-molecule ligands) to the outer layer of liposomes, enabling precise regulation of their physicochemical properties and biological behaviors. Thus, it plays a crucial role in enhancing physicochemical stability and cargo protective effects, improving mucus penetration, enabling active targeted delivery, and increasing bioavailability. Surface modification is of great significance for oral liposomes, as it can significantly improve their structural integrity, protective efficacy, and targeting in the GI tract. Physical or chemical modification of the liposome surface (e.g., PEGylation, chitosan coating, or conjugation with vitamins or peptide ligands) enhances their resistance to the GI environment, prolongs *in vivo* circulation time, and achieves active targeting to specific intestinal sites (e.g., small intestine or colon). These modifications also promote the adhesion of liposomes to intestinal epithelial cells or increase absorption through receptor-mediated endocytosis, thereby improving the oral bioavailability and therapeutic efficacy of active ingredients (Figure 2) (Khan et al., 2020; Zhang et al., 2023).

To comprehensively evaluate the efficacy of these diverse modification strategies, the pharmacokinetic improvements of various orally administered liposomes are summarized in Table 1. Notably, the rational selection of surface modification materials is closely related to the intrinsic biopharmaceutical properties of the encapsulated cargo. Therefore, the Biopharmaceutics Classification System (BCS) class of each drug is also included in Table 1 to provide a comprehensive perspective on how specific formulation designs address respective oral delivery barriers (e.g., poor aqueous solubility for BCS II/IV drugs, or low membrane permeability for BCS III biologics).

**TABLE 1:** Pharmacokinetic parameters of orally administered liposomes with different surface modification strategies.

Modification strategy type	Drug name	BCS class	Carrier form (comparison group)	AUC	T_1/2_	Tmax	Cmax	MRT0–t	Increase in relative bioavailability (%)	References
Synthetic polymer modification										
PEG	Fraxetin	II/IV	1. PEG-Lip 2. Free Drug (FD)	PEG-Lip: 39.96 ± 2.48 FD: 9.03 ± 0.84 (µg/mL × h)	PEG-Lip: 7.42 ± 0.70 FD: 5.44 ± 0.61 (h)	PEG-Lip: 1.58 ± 0.3 FD: 1.13 ± 0.31 (µg/mL)	PEG-Lip: 7.69 ± 0.43 FD: 1.84 ± 0.24 (µg/mL)	PEG-Lip: 9.17 ± 1.21 FD: 6.03 ± 0.91 (h)	PEG-Lip vs. FD: +343%	Miao et al. (2021)
PEG	Isoquercitrin	IV	1. PEG-Lip 2. Free Drug (FD)	PEG-Lip: 103.13 ± 4.16 FD: 11.25 ± 0.75 (µg/mL × h)	PEG-Lip: 15.31 ± 1.41 FD: 2.58 ± 0.14 (h)	PEG-Lip: 3.00 FD: 1.00 (h)	PEG-Lip: 13.03 ± 1.31 FD: 6.21 ± 0.78 (µg/mL)	PEG-Lip: 14.08 ± 1.67 FD: 10.99 ± 2.27 (h)	PEG-Lip vs. FD: +820%	Sheng et al. (2024)
TPGS	Syringic acid	IV	1. TPGS-Lip 2. Free Drug (FD)	TPGS-Lip: 338.08 ± 3.65 FD: 120.58 ± 2.92 (µg/mL × min)	TPGS-Lip: 50.03 ± 15.29 FD: 17.67 ± 0.93 (min)	TPGS-Lip: 8.00 FD: 8.00 (min)	TPGS-Lip: 4.50 ± 0.04 FD: 4.50 ± 0.04 (μg/mL)	TPGS-Lip: 82.76 ± 2.66 FD: 39.30 ± 1.01 (min)	TPGS-Lip vs. FD: +180%	Liu et al. (2019)
TPGS	Bisdemethoxycurcumin	IV	1. TPGS-Lip 2. Free Drug (FD)	TPGS-Lip: 6.06 ± 1.18 FD: 0.58 ± 0.18 (mg/L × h)	TPGS-Lip: 5.7 ± 0.98 FD: 1.512 ± 0.34 (h)	TPGS-Lip: 0.5 ± 0 FD: 0.17 ± 0 (h)	TPGS-Lip: 1.38 ± 0.37 FD: 0.4 ± 0.08 (mg/L)	TPGS-Lip: 9.46 ± 1.4 FD: 3.36 ± 0.26 (h)	TPGS-Lip vs. FD: +960%	Wang et al. (2021a)
TPGS	Nintedanib esylate	II	1. TPGS-Lip 2. Free Drug (FD)	TPGS-Lip: 12,138.75 ± 188.3 FD: 1945.88 ± 87.26 (ng/mL × h)	TPGS-Lip: 5.94 ± 0.97 FD: 2.24 ± 0.57 (h)	N/A	TPGS-Lip: 1,214 ± 72.74 FD: 501 ± 42.48 (h)	N/A	TPGS-Lip vs. FD: +523%	Kala and Chinni (2022)
TPGS	Chlorogenic Acid	III	1. TPGS-Lip 2. Free Drug (FD)	TPGS-Lip: 423.87 ± 34.6 FD: 284.51 ± 26.4 (ng/mL × h)	TPGS-Lip: 2.53 ± 0.7 FD: 1.58 ± 0.23 (h)	TPGS-Lip: 0.5 ± 0 FD: 0.5 ± 0 (h)	TPGS-Lip: 100.75 ± 10.3 FD: 110.44 ± 9.2 (ng/mL)	TPGS-Lip: 3.77 ± 0.22 FD: 2.47 ± 0.12 (h)	TPGS-Lip vs. FD: +52%	Zhang et al. (2024)
TPGS	6-Shogaol	II/IV	1. TPGS-Lip 2. Unmodified Liposomes (UL) 3. Free Drug (FD)	TPGS-Lip: 2,220.41 ± 24.21 UL: 1,077.79 ± 45.86 FD: 382.80 ± 47.24 (µg/mL × min)	TPGS-Lip: 364.91 ± 28.24 UL: 289.08 ± 27.6 FD: 195.02 ± 55.09 (min)	TPGS-Lip: 60 UL: 60 FD: 30 (min)	TPGS-Lip: 5.09 ± 0.24 UL: 4.04 ± 0.15 FD: 2.23 ± 0.16 (μg/mL)	TPGS-Lip: 393.00 ± 9.25 UL: 320.63 ± 24.56 FD: 216.05 ± 60.04 (min)	TPGS-Lip vs. FD: +480% TPGS-Lip vs. UL: +106%	Bao et al. (2020)
TPGS	Puerarin	IV	1. TPGS-Lip 2. Free Drug (FD)	TPGS-Lip: 25.69 ± 2.85 FD: 3.80 ± 0.20 (mg/mL × h)	TPGS-Lip: 10.21 ± 0.86 FD: 1.93 ± 0.26 (h)	TPGS-Lip: 2 FD: 0.75 (h)	TPGS-Lip: 4.58 ± 0.66 FD: 2.77 ± 0.29 (mg/mL)	TPGS-Lip: 10.42 ± 1.84 FD: 8.03 ± 3.71 (h)	TPGS-Lip vs. FD: +576%	Wang et al. (2024)
TPGS	Myricetin	IV	1. TPGS-Lip 2. Unmodified Liposomes (UL) 3. Free Drug (FD)	TPGS-Lip: 117.65 ± 2.48 UL: 59.17 ± 0.34 FD: 14.68 ± 0.56 (µg/mL × h)	TPGS-Lip: 7.15 ± 2.46 UL: 4.99 ± 0.11 FD: 0.98 ± 0.52 (h)	TPGS-Lip: 2 ± 0 UL: 2 ± 0.35 FD: 30 (h)	TPGS-Lip: 14.88 ± 1.53 UL: 7.56 ± 5.40 FD: 2.39 ± 0.6 (μg/mL)	TPGS-Lip: 11.37 ± 2.19 UL: 11.87 ± 0.13 FD: 6.07 ± 1.52 (h)	TPGS-Lip vs. FD: +701% TPGS-Lip vs. UL: +99%	Thant et al. (2021)
PMO	Idebenone	II	1. PMO-Lip 2. Unmodified Liposomes (UL) 3. Free Drug (FD)	PMO-Lip: 2047.00 ± 275.65 UL: 879.55 ± 124.50 FD: 539.88 ± 201.16 (ng/mL × h)	PMO-Lip: 6.97 ± 4.64 UL: 4.39 ± 4.11 FD: 3.69 ± 2.50 (h)	PMO-Lip: 0.17 ± 0.00 UL: 0.33 ± 0.19 FD: 2.25 ± 2.02 (h)	PMO-Lip: 1,153.75 ± 225.06 UL: 377.93 ± 99.92 FD: 81.84 ± 7.49 (ng/mL)	N/A	PMO-Lip vs. FD: +279% PMO-Lip vs. UL: +133%	Qian et al. (2022)
Biomacromolecule modification										
CS	Rivaroxaban	II	1. CS-Lip 2. Free Drug (FD) (Fed)	CS-Lip: 3,042.58 FD: 1889.32 (ng/mL × h)	CS-Lip: 4.58 FD: 4.58 (h)	N/A	CS-Lip: 1,045.22 FD: 931.07 (ng/mL)	N/A	CS-Lip vs. FD: +60%	Elsayad et al. (2021)
CS-TBA + PEG	Amikacin Sulfate	III	1. Composite: CS-TBA-PEG-Lip (CM) 2. CS-Lip 3. PEG-Lip	CM: 1726.14 ± 407.51 CS-Lip: 629.93 ± 404.68 PEG-Lip: 125.85 ± 53.96 (µg/mL × h)	CM: 2.76 ± 0.52 CS-Lip: 1.46 ± 1.16 PEG-Lip: 0.23 ± 0.00 (h)	CM: 2 CS-Lip: 1 PEG-Lip: 0.5 (h)	CM: 336 ± 50.12 CS-Lip: 233.33 ± 84.32 PEG-Lip: 106.67 ± 15.14 (µg/mL)	CM: 4.28 ± 0.49 CS-Lip: 2.51 ± 1.25 PEG-Lip: 0.86 ± 0.31 (h)	CM vs. CS-Lip: +450% CM vs. PEG-Lip: +1,560%	El-Say et al. (2024)
CA-CS-TGA	Azathioprine	IV	1. CA-CS-TGA-Lip 2. Unmodified Liposomes (UL) 3. Free Drug (FD)	CA-CS-TGA-Lip: 244.20 UL: 166.84 FD: 58.17 (µg/mL × h)	CA-CS-TGA-Lip: 8.17 UL: 8.17 FD: 1.5 (h)	CA-CS-TGA-Lip: 3.00 UL: 3.00 FD: 1.00 (h)	CA-CS-TGA-Lip: 22.54 UL: 15.64 FD: 8.65 (µg/mL)	N/A	CA-CS-TGA-Lip vs. FD: +320% CA-CS-TGA-Lip vs. UL: +46%	Arshad et al. (2024)
MCS	Andrographolide	II/IV	1. MCS-Lip 2. Unmodified Liposomes (UL) 3. Free Drug (FD)	MCS-Lip: 2,213.46 ± 50.05 UL: 1829.97 ± 141.66 FD: 1,410.3 ± 84.40 (ng/mL × h)	MCS-Lip: 16.17 ± 4.36 UL: 4.23 ± 1.13 FD: 4.06 ± 1.10 (h)	MCS-Lip: 2.00 ± 0.00 UL: 1.5 ± 0.86 FD: 1.33 ± 0.57 (h)	MCS-Lip: 495.90 ± 15.78 UL: 310.03 ± 12.64 FD: 207.14 ± 35.59 (ng/mL)	MCS-Lip: 19.43 ± 4.84 UL: 5.93 ± 1.53 FD: 8.6 ± 0.66 (h)	MCS-Lip vs. FD: +57% MCS-Lip vs. UL: +21%	Metkar et al. (2023)
GC	Ledipasvir	II	1. GC-Lip 2. Free Drug (FD)	GC-Lip: 88855.00 FD: 25994.10 (ng/g × h)	GC-Lip: 32.00 FD: 18.98 (h)	GC-Lip: 3.00 FD: 3.00 (h)	GC-Lip: 11400.00 FD: 3,420.00 (ng/g)	GC-Lip: 18.11 FD: 13.45 (h)	GC-Lip vs. FD: +240%	Fatouh et al. (2021)
NSCF	Paclitaxel	IV	1. NSCF-Lip 2. NSC-Lip 3. Unmodified Liposomes (UL)	NSCF-Lip: 8,981.44 NSC-Lip: 5,651.05 UL: 1,271.18 (ng/mL × h)	N/A	NSCF-Lip: 3.00 NSC-Lip: 5.00 UL: 1.00 (h)	NSCF-Lip: 866.61 NSC-Lip: 542.77 UL: 334.97 (ng/mL)	NSCF-Lip: 17.33 NSC -Lip: 23.22 UL: 5.60 (h)	NSCF-Lip vs. UL: +606% NSCF-Lip vs. NSC-Lip: +59%	Xing et al. (2024)
TAU-CS	Doxorubicin hydrochloride	III	1. TAU-CS-Lip 2. Unmodified Liposomes (UL) 3. Free Drug (FD)	TAU-CS-Lip: 4.69 UL: 2.67 FD: 1.36 (μg/mL × h)	TAU-CS-Lip: 24.23 UL: 3.64 FD: 2.12 (h)	TAU-CS-Lip: 2 UL: 8 FD: 8 (h)	TAU-CS-Lip: 0.48 UL: 0.36 FD: 0.26 (μg/mL)	TAU-CS-Lip: 5.93 UL: 7.56 FD: 2.89 (h)	TAU-CS-Lip vs. FD: +242% TAU-CS-Lip vs. UL: +74%	Qin et al. (2024)
Fruit Fiber	Berberine	IV	1. Fiber Interlaced Lip(FIL) 2. Free Drug (FD)	FIL: 1.38 ± 0.55 FD: 0.41 ± 0.04 (ng/mL × h)	FIL: 7.90 ± 1.58 FD: 7.32 ± 0.61 (h)	FIL: 0.25 ± 0.00 FD: 0.25 ± 0.00 (h)	FIL: 50.98 ± 27.03 FD: 15.54 ± 9.66 (ng/mL)	N/A	FIL vs. FD: +237%	Sharma et al. (2024)
CS + SA	Hydroxy-α-Sanshool	II/IV	1. Composite: CS-SA-Lip (CM) 2. Unmodified Liposomes (UL) 3. Free Drug (FD)	CM: 4,532.57 ± 446.41 UL: 1,084.37 ± 134.27 FD: 1,046.68 ± 122.46 (ng/mL × h)	CM: 9.29 ± 0.75 UL: 1.85 ± 1.51 FD: 1.53 ± 0.36 (h)	CM: 2.00 ± 0.47 UL: 0.50 ± 0.13 FD: 0.50 ± 0.17 (h)	CM: 664.49 ± 39.19 UL: 555.26 ± 38.38 FD: 523.25 ± 26.94 (ng/mL)	CM: 6.10 ± 0.19 UL: 1.44 ± 0.13 FD: 1.36 ± 0.10 (h)	CM vs. FD: +360% CM vs. UL: +320%	Tan et al. (2023)
Small-molecule ligand modification										
Pc-AT	Liraglutide	III	1. Composite: Pc-AT-Lip (CM) 2. Pc-Lip 3. Free Drug (FD)	CM: 3,100.19 ± 562.58 Pc-Lip: 2087.12 ± 117.90 FD: 1,356.92 ± 76.55 (pg/mL × h)	N/A	CM: 6 Pc-Lip: 6 FD: 3 (h)	CM: 475.18 ± 94.31 Pc-Lip: 323.15 ± 67.08 FD: 261.85 ± 135.27 (pg/mL)	N/A	CM vs. FD: +129% CM vs. Pc-Lip: +48%	Ding et al. (2024)
CPP	FU002	III	1. CPP-Lip 2. Free Drug (FD)	CPP-Lip: 732.2 FD: 136.1 (%ID × min)	N/A	N/A	N/A	N/A	CPP-Lip vs. FD: +438%	Werner et al. (2024)
RGD + PEG	Follicle stimulating hormone	III	1. Composite: RGD-PEG-Lip (CM) 2. PEG-Lip 3. Unmodified Liposomes (UL)	CM: 2,202.20 ± 8.74 PEG-Lip: 1769.20 ± 8.46 UL: 1,488.10 ± 6.76 (ng/mL × h)	CM: 17.12 ± 2.74 PEG-Lip: 20.90 ± 2.21 UL: 32.82 ± 1.45 (h)	CM: 24.00 ± 0.00 PEG-Lip: 24.00 ± 0.00 UL: 12.00 ± 0.00 (h)	CM: 41.80 ± 8.74 PEG-Lip: 36.20 ± 5.74 UL: 26.72 ± 4.21 (ng/mL)	CM: 39.40 ± 8.74 PEG-Lip: 39.00 ± 2.78 UL: 53.70 ± 4.95 h (h)	CM vs. UL: +48% CM vs. PEG-Lip: +25%	Raut et al. (2023)
FA	Erastin	II/IV	1. FA Lip 2. Free Drug (FD)	FA-Lip: 24.51 ± 2.73 FD: 6.78 ± 0.94 (µg/mL × h)	N/A	FA-Lip: 0.75 FD: 0.75 (h)	FA-Lip: 2.01 ± 0.21 FD: 0.68 ± 0.09 (µg/mL)	FA-Lip: 5.87 ± 0.63 FD: 3.52 ± 0.21 (h)	FA-Lip vs. FD: +258%	Hong et al. (2025)
B12+PEG	Insulin	III	1. Composite: B12-PEG-Lip (CM) 2. PEG-Lip 3. Unmodified Liposomes (UL) 4. Free Drug (FD)	CM: 346.66 ± 20.12 PEG-Lip: 166.05 ± 15.4 UL: 136.9 ± 12.4 FD: 47.22 ± 7.94 (μIU/mL × h)	N/A	CM: 0.5 ± 0.32 PEG-Lip: 4 ± 0.12 UL: 0.25 ± 0.12 FD: 0.5 ± 0.21 (h)	CM: 108.87 ± 4.5 PEG-Lip: 8.41 ± 1.15 UL: 10.4 ± 2.2 FD: 6.55 ± 0.78 (μIU/mL)	N/A	CM vs. FD: +634% CM vs. UL: +153% CM vs. PEG-Lip: +109%	Sarhadi et al. (2022)
SC + MAN	Sodium acetate	I	1. Composite: SC-MN-Lip (CM) 2. Unmodified Liposomes (UL) 3. Free Drug (FD)	CM: 86895.11 UL: 33169.36 FD: 29853.13 (μg/mL × h)	CM: 15.58 UL: 3.39 FD: 2.85 (h)	CM: 30 UL: 30 FD: 15 (min)	CM: 180.31 ± 1.64 UL: 141.32 ± 2.44 FD: 146.07 ± 1.22 (μg/mL)	N/A	CM vs. FD: +191% CM vs. UL: +162%	Hou et al. (2025)
pHPMA + R8+DOCA	Lurasidone hydrochloride	II	1. Composite: pHPMA-R8-MN-Lip (CM) 2. Free Drug (FD) (Fasted)	CM: 690.74 ± 86.19 FD: 274.89 ± 30.11 (ng/mL × h)	CM: 5.14 ± 0.51 FD: 4.43 ± 0.94 (h)	CM: 4.0 FD: 1.0 (h)	CM: 76.81 ± 7.38 FD: 55.23 ± 11.24 (ng/mL)	N/A	CM vs. FD: +151%	Song et al. (2025)

Abbreviations: AUC, area under the plasma concentration-time curve; BCS, biopharmaceutics classification system; Cmax, maximum plasma concentration; FD, free drug; Lip, liposome; MRT, mean residence time; N/A, not available/not assessed; Tmax, time to reach Cmax; T_1_/_2_, elimination half-life; UL, unmodified liposome.

The BCS classifications (Class I–IV) for certain natural products and extracts are estimated based on their documented aqueous solubility and intestinal permeability.

### 2.1 Synthetic polymer modification

To clearly distinguish from naturally derived biomacromolecules (such as polysaccharides and proteins, which will be discussed in Section 2.2), this section focuses specifically on the application of synthetic polymers (e.g., polyethylene glycol and its derivatives, Eudragit, and polydopamine) for liposome surface modification. Synthetic polymer modification is one of the earliest and most widely used surface engineering methods for liposomes. By covalent binding or physical coating, functional polymers are introduced to the liposome surface, leveraging their steric hindrance, physicochemical responsiveness, and bioadhesive properties to significantly improve the physicochemical stability, cargo protective capabilities and delivery efficiency of liposomes in the complex GI environment. This strategy not only forms a protective barrier against acid and enzymatic degradation but also regulates the interaction between the carrier and the biological membrane interface, enhancing mucosal penetration and absorption performance (Cao et al., 2022).

#### 2.1.1 Polyethylene glycol (PEG) and its derivative modification

##### 2.1.1.1 PEG modification

PEG modification is one of the most commonly used and well-studied surface functionalization strategies for liposomes (Deodhar and Dash, 2018). PEG chains form a flexible hydrophilic layer on the liposome surface, which can effectively reduce interparticle aggregation, enhance colloidal stability, and prolong *in vivo* circulation time. More importantly, PEGylation can promote the oral absorption of liposomes through the intestinal lymphatic pathway, thereby bypassing first-pass metabolism and improving systemic exposure and bioavailability (Singh et al., 2020a).

As early as 1999, Takeuchi et al. first reported PEG2000-modified insulin liposomes (PEG-Lips). The results showed that the retention time of PEG-Lips in the small intestine (approximately 201 s) was significantly longer than that of unmodified liposomes (approximately 86 s), and their interaction with the intestinal mucosa was enhanced, thereby improving the oral absorption of insulin (Iwanaga et al., 1999). To quantify these improvements, the efficacy of surface modifications is typically evaluated using key pharmacokinetic (PK) parameters: the area under the plasma concentration-time curve (AUC), reflecting total systemic drug exposure; the maximum plasma concentration (Cmax); the time to reach Cmax (Tmax); the elimination half-life (T_1_/_2_); and the mean residence time (MRT). The enhancement in oral absorption is primarily expressed as the percentage increase in the relative AUC (or relative bioavailability) compared to a control formulation (e.g., free drug or unmodified liposomes), calculated based on dose-normalized AUC ratios. Although no oral pegylated liposome formulations have been approved for marketing to date, numerous preclinical studies have demonstrated that this strategy can significantly enhance transepithelial transport and *in vivo* exposure.

*Ex vivo* non-everted gut sac models have shown that pegylated liposomes improve intestinal permeability. Singh et al. reported that the apparent permeability coefficient (Papp) of the pegylated formulation was 3.04 × 10^−4^ cm min^−1^, significantly higher than that of plain liposomes (2.17 × 10^−4^ cm min^−1^) and drug suspensions (0.9 × 10^−4^ cm min^−1^), indicating that PEG facilitates liposome penetration through the mucus layer and promotes transintestinal wall transport. *In vitro* cellular data are consistent with this finding. Flow cytometry results showed that the uptake of rhodamine B-loaded pegylated liposomes in MCF-7 breast cancer cells was nearly twice that of plain liposomes (M1 events: 2,466 vs. 1,341), suggesting that PEG modification enhances the interaction and internalization of nanoparticles with cell membranes (Singh et al., 2020b). Multiple *in vivo* pharmacokinetic studies have further confirmed the improvement of circulation time and systemic exposure by PEG modification. For example, Miao et al. observed a 343% increase in bioavailability with pegylated liposomes in the fraxetin system (Miao et al., 2021); Sheng et al. reported that PEGylated long-circulating liposomes increased the AUC_0_– ∞ of isoquercitrin (IQ) from 11.25 ± 0.75 μg h·mL^−1^ to 103.13 ± 4.16 μg h·mL^−1^, prolonged the half-life from approximately 2.6 h–15.3 h, and maintained detectable blood drug concentrations at 48 h, whereas free drugs were undetectable after 12 h, demonstrating significantly prolonged circulation time and exposure. In terms of therapeutic efficacy, Sheng et al. also confirmed in an ovariectomized rat osteoporosis model that IQ-Lips significantly improved bone microstructure compared with free IQ (increased bone volume fraction, trabecular thickness, trabecular number, bone mineral density; decreased trabecular separation, structure model index) and alleviated oxidative stress by increasing superoxide dismutase and glutathione peroxidase activities and decreasing malondialdehyde levels, thereby improving treatment outcomes (Sheng et al., 2024). Additionally, Farooq et al. developed PEG-coated vitexin liposomes with an encapsulation efficiency of approximately 80%, and PEG modification significantly enhanced the physicochemical stability and sustained-release performance of the formulation, showing great potential in oral drug delivery (Farooq et al., 2022).

Notably, PEGylation is not universally effective for all oral liposomes. Nishioka et al. observed the opposite result in GI administration: PEGylation did not improve the oral bioavailability of midazolam (11.7% ± 2.0% in the PEG group vs. 16.2% ± 4.6% in the non-PEG group). The authors speculated that this might be due to PEG layer-induced liposome aggregation, which hinders intestinal absorption (Nishioka et al., 2025). This result reminds us that the actual effect of PEG modification is strongly influenced by the physicochemical properties of the drug itself and the formulation process, requiring tailored design and verification for specific drugs.

Another key influencing factor is the coverage density of PEG on the liposome surface, which determines the spatial conformation of surface polymer chains and subsequent biological behaviors. According to de Gennes’ polymer interface conformation theory, low-density PEG exists in a “mushroom” distribution, high-density PEG forms a “brush” conformation, and medium density results in a “mushroom-brush transition” conformation (de Gennes, 1980). Experimental evidence indicates that these three conformations exhibit distinct performances in mucus penetration, physicochemical stability, and biodistribution. Wu et al.'s work pointed out that when the PEG modification ratio is approximately 5%, the liposome surface presents a transitional conformation, which not only provides sufficient steric hindrance and hydration layer to resist entrapment by gastric mucus but also avoids chain entanglement or aggregation caused by high-density PEG. In their comparison, the diffusion capacity of 5% PEG-Lips in mucus was approximately 6 times that of 2% PEG-Lips (mushroom conformation) and 1.3 times that of 10% PEG-Lips (brush conformation). Meanwhile, the 5% formulation outperformed other conformations in vesicle physicochemical stability, gastric mucosal distribution, drug absorption, and therapeutic effect on gastric intestinal metaplasia (Wu et al., 2022). Complementarily, Yamazoe et al. observed that the “brush” conformation formed at higher PEG density (approximately 10%) also confers strong mucus penetration ability. Among different PEG densities (3%, 7%, 10%, 15%), 10% PEG-Lips achieved the highest blood drug concentration (using oral FITC-dextran 4,000 as an indicator). However, the researchers also noted that an increase in blood drug concentration does not necessarily equate to better therapeutic efficacy. Excessively high PEG density may adversely affect drug release kinetics or local interactions. Therefore, trade-offs must be made based on the required cargo protective effects of the target drug, action target, and administration purpose during formulation optimization (Yamazoe et al., 2021).

##### 2.1.1.2 PEG derivative modification

In addition to conventional PEG, several PEG derivatives have shown additional advantages in oral liposome modification. D-α-tocopheryl polyethylene glycol succinate (TPGS) is a class of PEG derivatives formed by ester linkage between the succinyl group of vitamin E and PEG, commonly used as a functional coating or emulsifier for liposomes. Its synergistic mechanisms in oral liposomes mainly include three aspects: first, improving the solubility and encapsulation of hydrophobic drugs, thereby enhancing initial release and bioavailability; second, forming a hydrophilic/flexible protective layer on the particle surface to enhance tolerance to acid and enzymatic degradation and prolong *in vivo* retention; third, inhibiting membrane transporters (e.g., P-gp) to reduce drug efflux and increase intracellular accumulation and systemic exposure (Wong et al., 2025). Based on these functions, TPGS has been widely applied and verified in liposomes of numerous poorly soluble natural products and small-molecule drugs.

Multiple pharmacokinetic studies have shown that TPGS modification can significantly improve AUC, half-life, and MRT of oral administration. For example, Liu et al. introduced TPGS into the liposome system of syringic acid, resulting in an 180% increase in SA bioavailability compared with conventional formulations, a prolongation of blood half-life from approximately 17.7 min–50.0 min, and a significant extension of MRT, indicating that TPGS helps delay clearance and maintain *in vivo* exposure (Liu et al., 2019). In the bisdemethoxycurcumin system, Wang et al. reported that TPGS-modified liposomes increased oral bioavailability by approximately 960%, prolonged half-life by 3.8 times, and extended MRT by 2.8 times (Wang et al., 2021a). Kala et al. also observed a 523% increase in bioavailability in the nintedanib esylate system (Kala and Chinni, 2022). Zhang et al. used TPGS-modified liposomes for oral delivery of chlorogenic acid (CGA), which improved bioavailability by approximately 52% compared with CGA solution, prolonged T_1_/_2_ from 1.58 h to 2.53 h, and significantly extended MRT (Zhang et al., 2024).

In addition to simply increasing AUC, TPGS can also alter the tissue distribution and barrier penetration ability of drugs, thereby affecting therapeutic targeting. After introducing TPGS into 6-shogaol liposomes, Bao et al. observed that bioavailability was increased by approximately 480% compared with free drugs and 106% compared with unmodified liposomes. Meanwhile, brain tissue accumulation was significantly increased (brain concentration at 1 h post-administration was 2.0 times that of unmodified liposomes and 4.62 times that of free drugs) (Bao et al., 2020). Wang et al. found that TPGS-modified puerarin liposomes (TPGS-Puerarin-liposomes) exhibited a 576% increase in bioavailability compared with puerarin solution, a prolongation of T_1_/_2_ from 1.93 h to 10.21 h, a higher maximum blood drug concentration (Cmax: 4.58 vs. 2.77 mg/mL), and a longer time to peak (Tmax: 2 h vs. 0.75 h). Furthermore, they achieved targeted delivery to bone tissue and exerted multi-pathway anti-osteoporosis effects (Wang et al., 2024).

Moreover, the synergistic effect can be further amplified when TPGS is combined with proliposome technology. Thant et al. combined TPGS with proliposome technology to enhance the oral bioavailability and hepatoprotective activity of myricetin (MRC). Compared with unmodified liposomes and free MRC solution, TPGS-modified proliposomes (MRC-TPGS-PL) exhibited bioavailability increases of approximately 99% and 701%, respectively. The Cmax of the MRC-TPGS-PL group (14.88 μg/mL) was much higher than that of unmodified liposomes (7.56 μg/mL) and free MRC solution (2.39 μg/mL), and the half-life of the MRC-TPGS-PL group (7.15 h) was significantly longer than that of the free MRC group (0.98 h) (Thant et al., 2021).

TPGS also exerts a positive effect on the GI physicochemical stability and release behavior of formulations. Landi et al. used TPGS-modified liposomes (LPT-VD) for oral delivery of vitamin D (VD). In dissolution tests simulating the GI environment, the cumulative release of LPT-VD reached complete dissolution after 48 h, while the release of plain liposomes was only 77.68%. Additionally, the cellular uptake of the LPT-6C group (containing TPGS) was higher than that of the LP-6C group (without TPGS), indicating that TPGS significantly promotes the potential for intestinal cell absorption of vitamin D (Landi et al., 2025).

#### 2.1.2 Acrylic resin modification

Acrylic copolymers are characterized by significant pH responsiveness, with their molecular structure undergoing swelling or dissolution changes in response to environmental pH fluctuations. In the acidic gastric environment (pH < 5.5), cationic Eudragit E series or some neutral polymers remain insoluble, forming a stable physical barrier that effectively protects encapsulated drugs from degradation by gastric acid and digestive enzymes. When the formulation enters the neutral or weakly alkaline intestinal environment (pH > 6.0 or >7.0, depending on the Eudragit grade), anionic Eudragit L and S series undergo rapid deprotonation, swelling, and dissolution, enabling site-specific drug release in the small intestine or colon. This precise pH-triggered release property makes Eudragit an ideal polymer material for developing oral targeted drug delivery systems, especially colon-targeted and enteric-coated formulations, demonstrating great potential in improving drug bioavailability, achieving local therapy, and reducing systemic side effects (Nikam et al., 2023).

Existing studies have conducted extensive explorations on the physicochemical stability, cargo protective capabilities, and bioavailability enhancement of Eudragit-coated liposomes. Alizadeh et al. used Eudragit-coated liposomes (Eu-liposomes) for oral delivery of Salvia macilenta extract, significantly increasing the cellular uptake rate of marker components by approximately 20 times compared with free extracts (Alizadeh et al., 2025). Tomassi et al. coated liposomes with Eudragit L100 for delivery of fermented flour extract (Lisosan G), and the results showed that the coating significantly improved the physicochemical stability of liposomes under acidic gastric conditions while maintaining cellular safety (Tomassi et al., 2023). Similarly, Pani et al. coated cationic liposomes with Eudragit L100 to achieve efficient encapsulation and protected delivery of Ptilostemon casabonae extract. Only 10 mg/kg of Eudragit-modified liposomes achieved hypoglycemic effects comparable to 40 mg/kg of free extract (Pani et al., 2024).

On the other hand, researchers have also attempted to further enhance the targeting and functionality of Eudragit-coated liposomes through composite or multi-layer structures. De Leo et al. constructed PEG/Eudragit double-layer coated liposomes, improving the *in vivo* cargo protection and antioxidant activity of curcumin and hydroxytyrosol (De Leo et al., 2023). Fan et al. conjugated probiotic *Escherichia coli* Nissle 1917 with chrysin liposomes and encapsulated them in Eudragit L100-55, forming a pH-responsive probiotic-liposome hybrid system (EcNPIN-L) that achieves dual effects of inflammation control and gut microbiota regulation (Fan et al., 2025). Alghurabi et al. introduced bile salts into liposomes and coated them with Eudragit S100, constructing a bile salt composite liposome system (ES-SG/LP) that prolongs drug retention and promotes colon targeting (Alghurabi et al., 2023).

#### 2.1.3 Polydopamine modification

**FIGURE 3:**
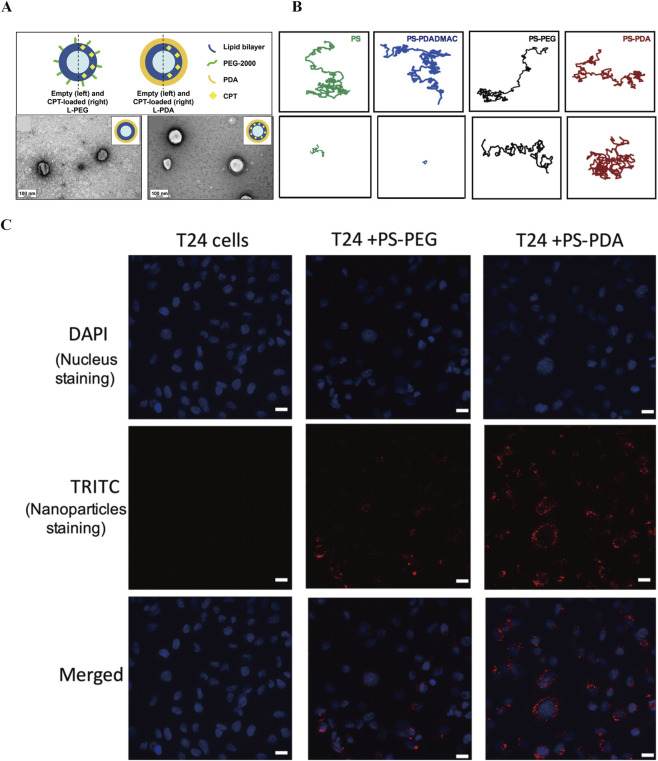
Structural characteristics, mucopenetration mechanisms, and cellular uptake outcomes of polydopamine (PDA)-coated nanocarriers. **(A)** Schematic representation and transmission electron microscopy (TEM) images of PDA-coated liposomes, showing the dense polymeric shell formed around the lipid vesicles. **(B)** Trajectory maps of nanoparticles tracking in water (top row) and reconstituted mucus (bottom row). Compared to bare particles (which are severely trapped), PDA-coated particles exhibit highly unhindered, Brownian-like diffusive trajectories in the mucus mesh, comparable to PEGylated particles. **(C)** Confocal Laser Scanning Microscopy (CLSM) images demonstrating the superior cellular uptake of PDA-coated particles (red fluorescence) in epithelial cells compared to PEG-coated counterparts after 24 h of incubation. **(A)** Adapted from Maurelli et al. (2024), licensed under CC BY. **(B, C)** Adapted with permission from Poinard et al. (2019), Copyright 2019 American Chemical Society.

Polydopamine (PDA) is a melanin-inspired synthetic biopolymer formed by the oxidative self-polymerization of dopamine monomers under alkaline conditions (Thirumalai et al., 2025). Rich in catechol functional groups, PDA exhibits excellent adhesive properties and versatile chemical reactivity, enabling the deposition of a dense and stable polymeric shell around lipid vesicles (Figure 3A) (Davidsen et al., 2021).

Poinard et al. reported that compared with PEG modification, PDA modification confers nanoparticles with excellent hydrophilicity and near-neutral/negative surface properties. This effectively reduces electrostatic and hydrophobic interactions with mucins, thereby significantly enhancing their mucus penetration ability to exhibit unhindered, Brownian-like diffusion trajectories in the mucus mesh (Figure 3B). Furthermore, the zwitterionic properties and interactions with cell membrane components brought about by the abundant phenolic hydroxyl and amino groups on the PDA surface significantly improve the cellular uptake efficiency of nanoparticles (Figure 3C). Therefore, PDA is considered a multifunctional nanoparticle surface modification strategy that combines efficient mucus penetration ability and strong cellular uptake promotion, holding the potential to replace traditional PEGylation strategies (Poinard et al., 2019). Maurelli et al. applied PDA coating to liposome systems loaded with the natural anticancer drug camptothecin. The results showed that PDA modification not only improved the physicochemical stability and drug-loading capacity of liposomes but also significantly enhanced mucus penetration and antitumor activity, especially exhibiting more than twice the cytotoxicity enhancement in three-dimensional (3D) tumor models (Maurelli et al., 2024).

Furthermore, owing to its robust surface reactivity, the PDA shell facilitates the facile secondary conjugation of diverse targeting ligands (e.g., specific aptamers, RGD peptides, or antibodies). This functionalization enables precise nanocarrier delivery and the integration of stimuli-responsive theranostic platforms—such as image-guided chemo-photothermal combined therapy—for treating various malignancies, including colorectal cancer. Although these advanced theranostic applications are currently explored predominantly *via* intravenous routes, they provide highly inspiring perspectives for oral delivery. By leveraging PDA’s highly functionalizable surface and inherent photothermal properties, future oral liposomal systems could be engineered to precisely target localized gastrointestinal lesions (e.g., orthotopic colorectal tumors) or achieve non-invasive theranostics through oral administration (Thirumalai et al., 2025).

#### 2.1.4 Poly (maleic anhydride-alt-1-octadecene) (PMO) modification

PMO contains multiple maleic anhydride groups that can chemically react with proteins or amino acids (e.g., amino groups in cysteine) in intestinal mucus to form amide bonds, thereby enhancing the adhesion ability between liposomes and the GI mucosa. Qian et al. constructed bioadhesive liposomes (IBLs) based on PMO and confirmed the mechanism by which PMO endows liposomes with bioadhesiveness through covalent bonding between its maleic anhydride groups and amino groups in intestinal mucus, thereby enhancing intestinal mucosal retention and significantly improving the oral bioavailability of idebenone (IDB). At 2 h post-oral administration, the IDB concentrations in the duodenum, jejunum, and ileum of the IBL group were 2.69 times, 3.21 times, and 3.44 times higher than those of plain liposomes, respectively. Compared with plain liposomes and free drug suspensions, the relative bioavailability of IBLs was increased by approximately 133% and 279%, respectively (Qian et al., 2022).

### 2.2 Biomacromolecule modification

Biomacromolecule modification involves modifying the liposome surface with natural or recombinant biomacromolecules (polysaccharides, proteins, peptides, *etc.*) as ligands. Such modifications not only possess good biocompatibility but also enhance the adhesion ability of liposomes to the GI mucosa through specific interactions and regulate their interactions with biological barriers (Nguyen et al., 2016).

#### 2.2.1 Natural polysaccharides

##### 2.2.1.1 Chitosan and its derivatives

Chitosan-coated oral liposomes offer multiple advantages: the cationic nature of chitosan allows it to form a physicochemically stable coating with the negatively charged liposome surface through electrostatic interactions, significantly enhancing colloidal stability and reducing drug leakage; its excellent mucoadhesive properties prolong the retention time of the formulation in the GI tract through electrostatic and hydrogen bonding interactions with mucins, promoting drug absorption; meanwhile, chitosan coating can protect liposomes from degradation by GI enzymes and damage by acidic environments, improving oral bioavailability. Additionally, controlled release or targeted delivery can be achieved by adjusting surface charge or functional modification, thereby enhancing therapeutic effects (Alcantara et al., 2025).

Natural chitosan-coated liposomes have shown good application potential in oral drug delivery systems. Using L-citrulline as a model drug, chitosan coating converted the zeta potential of liposomes from −65 mV to +64 mV, enhanced electrostatic repulsion between particles, improved colloidal stability, and increased encapsulation efficiency from 53.7% to 73.4%, significantly prolonging drug release time (Mohseni et al., 2025). Similarly, chitosan-caged liposomes developed by Elsayad et al. improved the bioavailability of rivaroxaban by approximately 60% and 27% under fed and fasted conditions, respectively (Elsayad et al., 2021). Chitosan-coated liposomes for oral delivery of piperine developed by Imam et al. exhibited an adhesion efficiency 2.8 times that of the uncoated group, a permeability flux 3.6 times higher than that of pure drugs, and enhanced anticancer activity against MCF-7 cells (Imam et al., 2021). Other studies have also confirmed that chitosan coating can improve the oral bioavailability of poorly soluble drugs such as vinorelbine, and its concentration has a significant impact on encapsulation efficiency, particle size, and physicochemical stability (Li et al., 2022; Guo et al., 2022). In more complex systems, Niu et al. combined chitosan-coated liposomes with *Clostridium* butyricum spores to achieve colon-specific targeting (Niu et al., 2024).

Furthermore, the natural antibacterial activity of chitosan can further enhance the therapeutic effects of liposomes. Gil-Gonzalo et al. found that 0.3% chitosan could inhibit the growth of *E. coli*; its coated ciprofloxacin liposomes significantly enhanced antibacterial effects through an integrated mechanism of “carrier as drug” (Gil-Gonzalo et al., 2024). De Souza et al. pointed out that positively charged chitosan electrostatically binds to the negative charge on the bacterial surface, promoting drug accumulation and disrupting the cell wall, thereby synergistically enhancing antibiofilm and antibacterial activities (de Souza et al., 2024).

Chemical modification of chitosan derivatives further expands their application potential in oral liposomes. Thiolated chitosan (Chitosan-N-acetylcysteine, Cs-NAC) is a derivative with thiol groups (–SH) introduced into the chitosan backbone, which can form disulfide bonds with mucins in intestinal mucus, thereby significantly enhancing the mucoadhesiveness of liposomes. As a glutathione precursor, NAC itself also has antioxidant activity, which complements the free radical scavenging ability of chitosan. Selmani et al. covalently immobilized thiolated chitosan on the liposome surface to construct a local drug delivery system that slowly releases selenium in the intestinal mucus layer (Selmani et al., 2022). This system can tightly adhere to the mucus layer without penetrating the intestinal epithelium, thereby improving safety and reducing non-specific uptake. Additionally, thiomers can interact with thiol groups of P-gp proteins, inducing conformational changes and blocking drug efflux function. El-Say et al. combined PEGylation with thiolated chitosan: PEGylation helps the carrier cross the mucus layer, while positively charged thiolated chitosan enhances mucoadhesion and locally inhibits P-gp, thereby significantly improving the intestinal absorption rate of amikacin. Compared with plain chitosan or PEG liposomes, the bioavailability of thiolated chitosan hybrid liposomes was increased by approximately 450% and 1,560%, respectively (El-Say et al., 2024). Arshad et al. further modified chitosan with both cholic acid (CA) and thiol groups to construct cholic acid-grafted thiolated chitosan (CA-CS-TGA). Cholic acid can specifically bind to the apical sodium-dependent bile acid transporter (ASBT) in the terminal ileum, synergizing with the adhesive effect of thiol groups to achieve a dual mechanism of “active transport + adhesion enhancement”. After coating azathioprine (AZA) with this carrier, the oral bioavailability was increased by approximately 46% and 320% compared with uncoated liposomes and pure drug suspensions, respectively (Arshad et al., 2024).

In addition to thiolation modification, glycosylated chitosan derivatives can endow liposomes with tissue targeting capabilities by introducing specific sugar ligands. Mannosylated chitosan (MCS) can recognize mannose receptors widely expressed in the intestines and liver, promoting receptor-mediated endocytosis. Metkar et al. used it to coat andrographolide liposomes, and the results showed that compared with unmodified carriers and pure drugs, the bioavailability was increased by approximately 21% and 57%, respectively, and the mean residence time was significantly prolonged (Metkar et al., 2023). Similarly, galactosylated chitosan (GC) achieves liver-targeted delivery by binding to asialoglycoprotein receptors on the surface of hepatocytes. Fatouh et al. reported that oral administration of GC-coated ledipasvir liposomes increased the liver AUC by approximately 240%, and Cmax and t½ were also significantly prolonged, showing excellent liver drug delivery potential (Fatouh et al., 2021).

Furthermore, Xing et al. developed a chitosan derivative (NSCF) co-modified with N-succinic anhydride and D-fructose for modifying paclitaxel (PTX) polymer liposomes. N-succinic anhydride can target intestinal epithelial cell monocarboxylate transporters, while fructose can recognize glucose transporters, achieving multi-ligand synergistic absorption and breaking through the transport saturation limitation of a single ligand. Compared with unmodified and only N-succinic anhydride liposomes, the oral bioavailability of this system was increased by approximately 606% and 59%, respectively, showing a significant intestinal absorption promotion effect (Xing et al., 2024). Qin et al. constructed a taurine-chitosan conjugate (TAU-CS) for surface coating of liposomes. This system significantly enhanced the intestinal absorption of doxorubicin hydrochloride (DOX·HCl)-loaded liposomes through specific recognition between taurine and intestinal TAUT transporters. Compared with uncoated liposomes (LIP@DOX·HCl), free DOX·HCl solution, and competitive inhibition groups containing free taurine (TAU-CS/LIP@DOX·HCl + Free TAU), the oral bioavailability of TAU-CS-coated liposomes (TAU-CS/LIP@DOX·HCl) was increased by approximately 242%, 74%, and 31%, respectively (Qin et al., 2024).

##### 2.2.1.2 Hyaluronic acid (HA)

HA is a naturally derived linear glycosaminoglycan with excellent biocompatibility, biodegradability, low toxicity, and non-immunogenicity, thus being widely used in liposome surface modification (Ni et al., 2025).

Sun et al. used HA as a coating material to prepare nanoliposomes (F-HA-NLs) loaded with the flavonoid active ingredient fisetin. The study systematically investigated the effects of HA with different molecular weights (3–1,500 kDa) and concentrations (0.1%–1.5%) on liposome properties. The results showed that HA with a molecular weight of 35 kDa at a concentration of 0.4% achieved the best overall performance. HA coating significantly improved the physicochemical stability of liposomes and their cargo protective capabilities during digestion, increasing the bioaccessibility of fisetin in F-HA-NLs by approximately 4% and 1,185% compared with plain liposomes and free fisetin, respectively (92.5% vs. 88.9% vs. 7.2%) (Sun et al., 2024).

Additionally, HA can specifically bind to CD44 receptors highly expressed on the surface of inflammatory cells, tumor cells, and intestinal epithelial cells, thereby achieving active targeted delivery. Chen et al. utilized the high affinity of HA for CD44 receptors, and combined it with reactive oxygen species-responsive ketothiol to construct nanoparticles (Ra@TH) loaded with rapamycin. Compared with free rapamycin, Ra@TH exhibited higher drug accumulation and local concentration in colonic inflammatory tissues, significantly enhancing anti-inflammatory therapeutic effects (Chen et al., 2024). In addition to serving as a single targeting ligand, HA can synergize with other functional materials to construct more complex intelligent delivery systems. For example, Wang et al. constructed a dual-targeted liposome system for colon cancer treatment. In this system, HA is responsible for actively recognizing and enriching in tumor cells with high CD44 receptor expression, while inulin, as a prebiotic specifically degraded by gut microbiota, endows liposomes with colon enzyme-responsive drug release capabilities. Through the synergy of active targeting by HA and lesion retention by inulin, this dual-modified liposome showed significantly improved uptake efficiency in the C26 colon cancer cell model (the uptake efficiency of FITC-labeled drugs and Ce6 was increased by approximately 135% and 633% compared with free drugs, respectively) and exhibited superior intestinal mucosal targeting and antitumor efficacy (Wang et al., 2025).

##### 2.2.1.3 Pectin

Pectin is a natural, safe anionic polysaccharide widely present in citrus fruits and apples, with good biocompatibility, degradability, and various health benefits (e.g., regulating gut microbiota, anti-inflammatory, antidiabetic). In the food and pharmaceutical fields, pectin is often used to modify the liposome surface to improve its physicochemical stability, cargo protective capabilities and oral delivery performance (Ni et al., 2025).

Lutta et al. coated cationic liposomes with pectin *via* electrostatic adsorption using microfluidic technology and systematically evaluated the key parameters affecting the coating effect. The results confirmed that a stable system with uniform particle size and significantly negative-shifted surface charge was obtained when the pectin/liposome mass ratio was 0.7 and the flow rate ratio was 2:1. Further comparison of different pectin structures revealed that low-methoxylated pectin (LM-pectin) and low-methoxylated amidated pectin (LMA-pectin) could form denser and more stable coating layers, while high-methoxylated pectin (HM-pectin) exhibited limited coating effects due to weak electrostatic interactions (Lutta et al., 2025).

Additionally, Su et al. coated liposomes with pectin (P-lips) and chitosan (C-lips) respectively for delivering phenylethanoid glycosides, comparing their physicochemical stability, cargo protective capabilities, and cellular activity in simulated intestinal fluid. The results showed that the drug retention rates of both P-lips and C-lips were approximately 75%, 25% higher than that of plain liposomes. In HSC-T6 hepatic stellate cells, P-lips exhibited the lowest IC_50_ (180.76 μg/mL) and the strongest inhibitory activity, indicating that pectin coating helps enhance the cargo protective effects and efficacy of drugs (Su et al., 2024a).

In addition to using purified pectin directly, natural fruit fibers rich in pectin and other cell wall polysaccharides have also been developed as unique liposome surface modification materials. Sharma et al. used natural fruit fiber as a surface modification material to form a “fiber wall” structure on the liposome surface through electrostatic interactions with phospholipids (lecithin) for oral delivery of berberine (BBR). The results showed that this “fiber interlaced liposome” significantly increased the *in vivo* exposure of BBR, with bioavailability 237% higher than that of free drugs and Cmax increased from 15.54 ng/mL to 50.98 ng/mL, indicating that pectin-based materials can effectively enhance oral absorption (Sharma et al., 2024).

##### 2.2.1.4 Sodium alginate (SA) modification

SA is a naturally occurring anionic linear polysaccharide with advantages such as inherent chemical stability, high solubility, high viscosity, safety, and ease of production, making it an excellent coating material (Duan et al., 2023).

Wu et al. used SA as a coating material to modify the liposome surface *via* physical adsorption, forming a dense outer barrier. This coating not only significantly improved the storage stability of liposomes but also enhanced their tolerance in the GI environment and transcellular permeability. Experimental results showed that compared with uncoated liposomes, SA-coated collagen peptide liposomes maintained good physicochemical stability after 1 month of storage, with GI physicochemical stability and cargo protection effects (as indicated by bioavailability) enhanced by approximately 50%, transcellular permeability enhanced by 18%, and *in vitro* drug release rate reduced by approximately 34%. This indicates that SA modification can significantly improve oral absorption performance while delaying drug release (Wu et al., 2023).

##### 2.2.1.5 Gum Arabic (GA) modification

GA is a complex natural polysaccharide biopolymer with excellent water solubility, emulsifying properties, and surface activity, commonly used for interface stabilization of drug delivery systems. GA can bind to the liposome surface through non-covalent interactions, improving its physicochemical stability, encapsulation efficiency, and antioxidant performance, while regulating drug release kinetics (Hudiyanti et al., 2024).

Cheng et al. encapsulated blueberry anthocyanins in liposomes and further coated them with GA to form a composite system (Anthocyanin–Liposome–GA complex). The results showed that this system significantly improved the antioxidant activity and cargo protection of anthocyanins, enhanced cellular uptake efficiency, and exhibited superior biological effects in improving liver lipid metabolism and regulating gut microbiota (Cheng et al., 2024a). Additionally, subsequent studies systematically compared the effects of four natural polysaccharides—GA, guar gum, pullulan, and xanthan gum—on liposome performance. The results indicated that GA-modified liposomes performed the best in maintaining liposome structural integrity, protecting anthocyanin activity, and improving biocompatibility (Cheng et al., 2024b).

##### 2.2.1.6 Composite polysaccharide coatings

**FIGURE 4:**
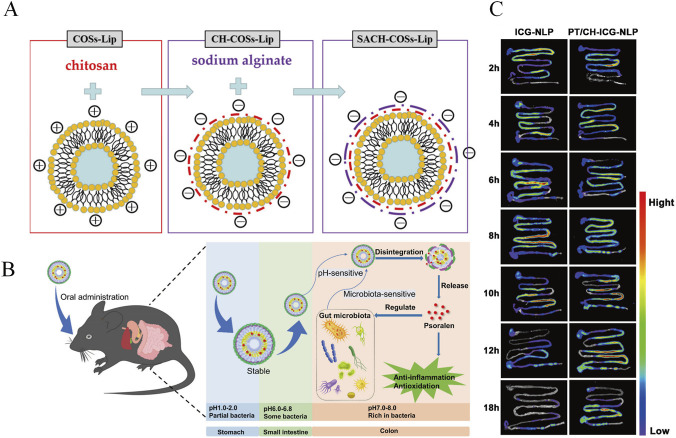
Process, mechanisms, and *in vivo* outcomes of layer-by-layer (LbL) polysaccharide-coated oral liposomes for targeted delivery. **(A)** Mechanistic illustration of the LbL self-assembly process, where cationic (e.g., chitosan) and anionic (e.g., sodium alginate) polysaccharides are sequentially coated onto the liposome surface *via* electrostatic interactions. **(B)** Schematic representation of the dual-responsive (pH and microbiota-sensitive) disintegration mechanism. The outer polysaccharide layer protects the liposomes in the harsh acidic environment of the upper gastrointestinal tract but is degraded by specific enzymes secreted by gut microbiota upon reaching the colon, triggering localized drug release. **(C)** *Ex vivo* fluorescence imaging demonstrating the superior colon-targeted delivery outcome. Compared to uncoated liposomes (which are prematurely destructed and rapidly cleared), the LbL-coated liposomes maintain structural integrity during transit and exhibit significant, prolonged fluorescence accumulation specifically in the colonic region. **(A)** Adapted from Cui et al. (2021), licensed under CC BY. **(B, C)** Adapted with permissio of the Royal Society of Chemistry form Su et al. (2024b), Copyright 2024 Royal Society of Chemistry; permission conveyed through Copyright Clearance Center, Inc.

Single-layer polysaccharide-modified liposomes still face problems such as vesicle aggregation and easy leakage of active substances. Constructing multi-layer polysaccharide composite coatings on the liposome surface through layer-by-layer (LbL) self-assembly or sequential electrostatic deposition technology (Figure 4A) can significantly improve their physicochemical stability, cargo protective capabilities and controlled release performance (Ni et al., 2025).

The pectin–chitosan double-layer system is one of the most representative composite modification structures. Wang et al. prepared liposomes with pectin and chitosan as double-layer coating materials for loading β-carotene and coix seed oil (Wang et al., 2023). This system exhibited slower and more controlled release behavior in simulated GI fluids. After testing with a complete simulated digestion model, the retention of β-carotene in double-layer coated liposomes was 34% higher than that in uncoated liposomes. Su et al. utilized LbL self-assembly technology to sequentially deposit positively charged chitosan (CH) and negatively charged pectin (PT) on the liposome surface, constructing a PT/CH double-layer coating structure. The outer pectin remains stable under low pH conditions in the stomach, while in the colon with higher pH, the electrostatic attraction between CH and PT weakens. Meanwhile, pectinase and glycosidase secreted by colonic anaerobic bacteria can specifically degrade the coating layer, triggering a dual-responsive (pH and microbiota-sensitive) “burst release” of drugs (Figure 4B). *Ex vivo* imaging further confirmed that these LbL-coated liposomes maintain structural integrity during transit and exhibit significant, prolonged accumulation specifically in the colonic region compared to uncoated liposomes (Figure 4C), successfully achieving colon-specific delivery (Su et al., 2024b). Similarly, Xian et al. constructed double-layer coated liposomes (Cel/PT-LbL Lipo) for delivering celastrol by introducing a pectin/trimethylated chitosan composite layer on the liposome surface. The results showed that this system significantly increased the local drug concentration and cellular uptake rate of celastrol in the colon, with the survival rate increased from 33.3% to 88.9% in animal experiments, effectively reducing toxicity (Xian et al., 2021).

Another typical structure is chitosan–sodium alginate double-layer modification. Dong et al. used SA and CS to co-modify fucoxanthin liposomes, and the *in vitro* bioaccessibility was increased by approximately 45.5% and 23.6% compared with unmodified and single-layer modified liposomes, respectively (Dong et al., 2023). Jang et al. significantly improved the cargo protective effects of curcumin in gastric acid and sustained intestinal release performance through a chitosan/alginate coated nano-liposome (Jang et al., 2024). Cui et al. sequentially coated chitosan and sodium alginate on the surface of chitooligosaccharide (COSs)-loaded liposomes through electrostatic interactions, significantly enhancing their oral physicochemical stability, anti-digestion ability, and intestinal absorption efficiency. The Papp was increased by 18.2% and 43.9% compared with single-layer and unmodified liposomes, respectively (Cui et al., 2021). Tan et al. adopted the same LbL technology with sodium alginate and chitosan as coating materials to prepare composite liposomes (SA/CH-HAS-LIP) coated with hydroxy-α-sanshool (HAS). The bioavailability was increased by approximately 360% and 320% compared with free HAS solution and uncoated liposomes, respectively (Tan et al., 2023). Hu et al. also modified apple peel polyphenol (APPs) liposomes (APPL) through LbL technology with CS and SA, constructing SA-CS-APPL double-layer coated liposomes, whose bioavailability was increased by 28% compared with free APPs (Hu et al., 2025). Additionally, Patır et al. used this strategy to double-coat Alpinia officinarum Hance extract-loaded liposomes, increasing the *in vitro* bioaccessibility of the main active ingredient galangin from approximately 24%–74% (Patir et al., 2025).

In addition to pectin and sodium alginate, other polysaccharide combinations have also been used for composite modification. Guan et al. constructed double-layer coated liposomes with CS and chondroitin sulfate for delivering betanin, effectively improving the physicochemical stability of the liposomes and the cargo protective effects for betanin in different pH, ionic strength, and GI environments and achieving sustained release (Guan et al., 2024). Ettoumi et al. used fucoidan (F) as the second layer to form an F/CS double-layer structure with chitosan for co-encapsulating hydrophilic catechin and hydrophobic juglone, improving the encapsulation efficiency, antioxidant activity, and controlled release performance of liposomes (Ettoumi et al., 2023).

#### 2.2.2 Protein and peptide modification

Introducing protein or peptide molecules with specific biological functions (e.g., adhesion proteins, enzymes, or cell-penetrating peptides (CPPs)) to the liposome surface can endow liposomes with properties such as mucus penetration, enhanced endocytosis, and active targeting. Protein modification relies on its inherent structural integrity and biocompatibility, while peptide modification has become a research hotspot due to its advantages of small molecular weight, ease of synthesis, and strong targeting (Liu et al., 2024).

##### 2.2.2.1 Protein modification

Traditional nanocarriers often struggle to simultaneously meet the requirements of mucus penetration and transmembrane absorption: mucus penetration requires a hydrophilic and electrically neutral surface to reduce adhesion to negatively charged mucins; while transintestinal epithelial absorption requires a positively charged or hydrophobic surface to promote interactions with negatively charged cell membranes and enhance endocytosis. Ding et al. designed an adaptive protein corona–AT1002–cationic liposome (Pc-AT-CLs) to address the contradiction between mucus penetration and transmembrane absorption. When crossing the mucus layer, the “protein corona” composed of bovine serum albumin (BSA) can reduce interactions with mucins, thereby enhancing mucus penetration. When liposomes reach the intestinal epithelium, the outer BSA is degraded by trypsin, exposing the internal positively charged cationic liposomes (AT-CLs), which further promote interactions and endocytosis with cell membranes (Ding et al., 2024). Meanwhile, the AT-1002 peptide can regulate tight junctions between cells, further enhancing absorption. The results showed that the mucus penetration of Pc-AT-CLs was 1.45 times that of AT-CLs, and the Papp of AT-CLs was 2.03 times higher than that of plain cationic liposomes. The overall bioavailability was increased by approximately 48% and 129% compared with BSA-coated liposomes and free liraglutide, respectively.

Park et al. covalently modified the liposome surface with the enzymatically active protein bromelain to construct bromelain-modified liposomes (Bro-Lip), achieving dual functions of mucus dissolution and enhanced absorption. The results showed that the penetration ability of Bro-Lip in the porcine intestinal mucus model was 2.87 times higher than that of unmodified liposomes, the Papp was increased by 3.59 times in the Caco-2 monolayer model, and 2.25 times in the Caco-2/HT29 co-culture model. Cellular uptake experiments further confirmed that the internalization efficiency of Bro-Lip was approximately 1.4 times higher than that of plain liposomes (Park et al., 2024). Additionally, Tan et al. modified liposomes with the surface layer protein B (SlpB) of the probiotic Levilactobacillus brevis JCM 1059, enabling active targeting to antigen-presenting cells (APCs) in Peyer’s patches of the intestines. This modification significantly improved the GI physicochemical stability and M cell transmembrane transport efficiency of liposomes. In the mouse model, the fluorescence signal intensity of SlpB-modified liposomes in the M cell region was 2.03 times that of the unmodified group, and they were efficiently taken up by APCs (CD23^+^ cells), demonstrating strong targeting and immune delivery potential (Tan et al., 2022).

##### 2.2.2.2 Peptide modification

Werner et al. prepared liposomes using archaeal tetraether lipids (GCTE) and modified their surface with CPPs for delivering FU002. The positive charge of CPPs can interact with the negatively charged intestinal mucosa, enhancing liposome adhesion, and promoting transepithelial transport through endocytosis or direct membrane penetration mechanisms. Compared with free drugs, the bioavailability of FU002 encapsulated in CPP-GCTE liposomes was significantly increased by approximately 438%, showing a significant promotion effect of CPPs in oral delivery (Werner et al., 2024). Among various CPPs, the cationic octaarginine (R8) has been extensively studied due to its strong positive charge properties. Deng et al. externally combined R8-PEG modified paclitaxel liposomes (R8-PEG@PLs) with a novel P-gp inhibitor (PgpI). R8-PEG modification significantly improved intestinal absorption of liposomes, while the synergistic effect of PgpI further inhibited drug efflux. The cellular internalization rate of this combination was 2.3 times that of unmodified liposomes, and the *in vivo* antitumor effect was increased by 2.2 times, significantly improving the oral bioavailability of paclitaxel (Deng et al., 2024). Wang et al. used selenium-substituted apamin (Se-apamin) as a targeting ligand, which can specifically recognize intestinal glial cells and central astrocytes. Meanwhile, a non-covalently cross-linked lactic acid chitooligosaccharide (COL) coating was introduced on the liposome surface to enhance its GI physicochemical stability and penetration. The results showed that the dual-modified curcumin (CUR)-loaded liposomes (C-SA-Lip/CUR) exhibited the highest oral absorption efficiency among different groups, and its effective permeability coefficient was increased by 4.6%, 46.8%, 145.9%, and 295.7% compared with COL-coated only, Se-apamin-modified only, plain liposomes, and free CUR solution, respectively (Wang X. et al., 2021). Raut et al. covalently linked the arginine–glycine–aspartic acid (RGD) tripeptide to the liposome surface to achieve active targeting to M cells in intestinal Peyer’s patches. The RGD peptide can recognize and bind to β_1_ integrins highly expressed on the apical membrane of M cells, significantly improving the oral bioavailability of follicle-stimulating hormone (FSH). Compared with PEG-modified liposomes and unmodified liposomes, the bioavailability of RGD-modified liposomes was increased by approximately 25% and 48%, respectively (Raut et al., 2023).

#### 2.2.3 Antibody modification

Antibody modification is based on the specific binding principle of antigens and antibodies. By conjugating monoclonal or polyclonal antibodies against antigens on the surface of target cells to the liposome surface, precise recognition and targeted enrichment of target cells or lesion areas are achieved. Wang et al. constructed an orally administered liposome modified with a monoclonal antibody against *H. pylori* (*Helicobacter pylori*, Hp Ab), achieving high-specificity recognition and targeting of *H. pylori* in the stomach. This liposome encapsulates the photosensitizer indocyanine green (ICG), which can generate reactive singlet oxygen (^1^O_2_) under ultrasound irradiation, thereby achieving sonodynamic killing of *H. pylori*. Additionally, ICG has photoacoustic signal properties, enabling real-time imaging monitoring during treatment and providing possibilities for precise treatment and integrated diagnostic imaging and treatment (Wang et al., 2022).

#### 2.2.4 Aptamer modification

Aptamers are single-stranded DNA or RNA fragments obtained through systematic evolution of ligands by exponential enrichment (SELEX) technology. They have binding specificity and affinity comparable to antibodies, along with advantages such as small molecular weight, high structural and nuclease stability, and ease of synthesis and modification (Maimaitiyiming et al., 2019). Using aptamers for liposome surface modification can promote active transmembrane transport and local enrichment of liposomes by recognizing specific receptors on intestinal epithelial cells (e.g., M cells) or target tissues, thereby significantly improving oral drug absorption and tissue targeting efficiency. He et al. obtained high-affinity and high-specificity M cell-targeted aptamers through cell-based SELEX and sequence truncation optimization, and prepared aptamer-modified liposomes (Apt-Lip-EXT) using exenatide (EXT) as a model drug. The study showed that compared with non-targeted liposomes (Lip-EXT), the cellular uptake rate of Apt-Lip-EXT was increased by approximately 97%, the *in vitro* transport efficiency was increased by approximately 95%, and absorption in small intestinal Peyer’s patches was significantly enhanced, indicating that aptamer modification can effectively promote M cell-mediated transport and targeted delivery of liposomes (He et al., 2023).

### 2.3 Small-molecule ligand modification

In oral liposome delivery systems, to overcome GI barriers and improve drug absorption efficiency, researchers have widely used small-molecule active substances as targeting ligands to modify the liposome surface for active targeting. These small-molecule ligands are usually natural physiological molecules or their analogs with molecular weights below 1,000 Da. By covalent conjugation or physical insertion, these ligands are anchored on the liposome surface, which can effectively “hijack” specific receptor-mediated endocytosis or carrier-mediated active transport pathways existing in the intestines, thereby significantly enhancing the transmembrane absorption ability of nanocarriers. Notably, unlike therapeutic cargos encapsulated within the internal aqueous core or embedded within the lipid bilayer, these targeting molecules (such as folic acid, biotin, and vitamins) are exclusively grafted onto the exterior surface of the liposomes. They do not exert pharmacological effects themselves; rather, these surface-exposed moieties function strictly as navigational “keys.” By specifically recognizing and binding to corresponding receptors overexpressed on target cellular barriers, they serve as the crucial structural foundation for the aforementioned active transport processes, ultimately ensuring the precise cellular uptake of the internal payload (Shreya et al., 2018).

#### 2.3.1 Organic acid modification

Studies have found that palmitic acid (PA)-modified liposomes can promote the absorption of liposomes by intestinal epithelial cells through the fatty acid transporter protein 4 (FATP4)-mediated pathway, and may enhance paracellular permeability by regulating the structure of tight junction proteins, thereby significantly improving the oral permeability of bioactive peptides. Using the tripeptide Val-Pro-Pro as a model drug, the intestinal permeability of PA-modified liposomes (P-LP) was increased by approximately 44% and 93% compared with unmodified liposomes (P-L) and free peptides, respectively. Meanwhile, in the Caco-2 cell model, the uptake efficiency of palmitic acid-modified blank liposomes (BLP) was increased by 191% and 296% compared with unmodified liposomes (BL) and free probes (Free C6), respectively (Huang et al., 2025).

#### 2.3.2 Folic acid modification

Folic acid (FA) receptors are highly expressed on intestinal epithelial cells (especially in the jejunum) and some lesion tissues (e.g., tumor cells, inflammation-activated macrophages). Therefore, folic acid can be used as an effective small-molecule targeting ligand for liposome modification to achieve active delivery (Kumar et al., 2019).

Yazdi et al. covalently linked folic acid to DSPE-PEG3400 and co-constructed PEG-Lip-FA liposomes with PEG-modified lipids (mPEG2000-DSPE) for oral delivery of insulin. The results showed that PEG-Lip-FA containing 1 mol% and 2 mol% folic acid exhibited significantly higher serum insulin peaks at 4 h post-oral administration than non-targeted and non-pegylated groups. Among them, PEG-Lip-FA 1% showed the strongest hypoglycemic effect, even exceeding subcutaneous insulin in the early stage of administration (one to two h) (Yazdi et al., 2020). Folic acid-modified liposomes can also be used for inflammation targeting. Li et al. designed FA-LP-GCK liposomes, which recognize activated macrophages with high folic acid receptor expression in rheumatoid arthritis lesions through folic acid and inhibit P-gp-mediated drug efflux through TPGS, thereby achieving oral delivery and improving targeting. *In vitro* experiments showed that the uptake of FA-LP-GCK by M1-type macrophages was at least twice that of non-targeted liposomes, significantly enhancing the therapeutic effect of drugs (Li et al., 2023). In terms of tumor targeting applications, Wang et al. constructed a FA-modified Hemesome-ART liposome system (FA-Hemesome-ART) for co-delivering artemisinin (ART) and hemin to enhance cancer chemotherapy and immunotherapy effects. The results showed that the Papp of FA-Hemesome-ART was increased by approximately 6 times compared with free ART, and AUC was significantly improved, showing efficient oral targeted delivery ability (Wang et al., 2021c). Similarly, Hong et al. synthesized folic acid-modified trifluorooctyl chain fluorinated amphiphilic molecules (FA-3F conjugate), which self-assembled into fluorinated liposomes (FA-3F-LS) for oral delivery of erastin. Compared with free erastin, the bioavailability of erastin@FA-3F-LS was increased by approximately 258%, Cmax was increased by approximately 271%, and the MRT was prolonged from approximately 66 min–903 min, further verifying the potential of folic acid in enhancing intestinal absorption and targeted delivery (Hong et al., 2025).

#### 2.3.3 Vitamin B12 modification

The receptor of vitamin B12, CD320, is highly expressed in the small intestine (Chen et al., 2022), Sarhadi et al. covalently linked vitamin B12 to the surface of pegylated liposomes to construct B12-targeted insulin liposomes (Lip-PEG-PB). Through receptor-mediated endocytosis and transcellular transport, this system significantly improved the absorption efficiency of liposomes in the intestines. Compared with non-targeted pegylated liposomes (Lip-PEG), unmodified liposomes, and free insulin solution, the oral bioavailability of B12-modified liposomes was increased by approximately 109%, 153%, and 634%, respectively, showing the significant advantage of vitamin B12 modification in enhancing oral liposome absorption (Sarhadi et al., 2022).

#### 2.3.4 Bile acid derivatives as targeting ligands

**FIGURE 5:**
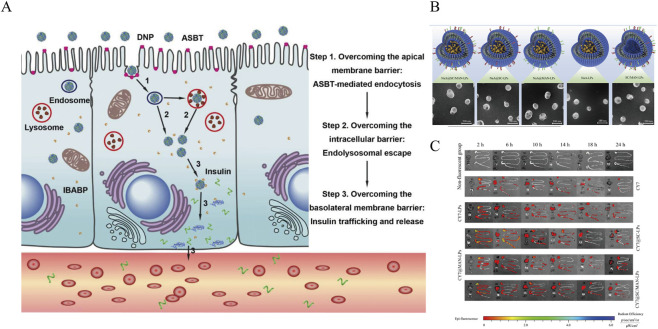
Transport mechanisms, structural characteristics, and *in vivo* outcomes of bile acid-modified liposomes utilizing endogenous transporter pathways. **(A)** Schematic illustration of the active transcytosis mechanism. Bile acid-decorated liposomes are efficiently internalized by the apical sodium-dependent bile acid transporter (ASBT), successfully escape from endolysosomal degradation, and are transcytosed across the intestinal epithelium into the systemic circulation. **(B)** Three-dimensional structural diagrams and corresponding transmission electron microscopy (TEM) images of dual-targeted liposomes modified with sodium cholate and mannose. **(C)** *Ex vivo* intestinal fluorescence imaging highlighting the dramatic improvement in structural stability and targeted tissue accumulation compared to non-targeted liposomes. **(A)** Reproduced with permission from Fan et al. (2018), Copyright 2017, with permission from Elsevier. **(B, C)** Adapted from Hou et al. (2025), licensed under CC BY.

Bile acids (BA) are naturally occurring amphiphilic molecules that play an important role in lipid digestion and absorption. Conventionally, free bile salts have been directly incorporated into the lipid membrane as structural components to form “bilosomes”—a strategy fundamentally classified as bilayer composition modification. However, recent advancements have alternatively harnessed bile acid derivatives exclusively as exterior-facing targeting ligands (Harini et al., 2025). In this context, they can be utilized as efficient small-molecule navigational moieties for modifying the surface of oral liposomes. The core of this strategy is to “hijack” the ASBT highly expressed in the terminal ileum. ASBT can efficiently mediate the active absorption of bile acids, and using this pathway can significantly improve the intestinal uptake efficiency and subsequent active transcytosis of liposomes (Figure 5A) (Fan et al., 2018).

Hou et al. developed an oral liposome system loaded with sodium acetate (NaA) targeting hepatocytes, Kupffer cells, and the intestines by modifying the liposome surface with sodium cholate (SC) and mannose (MAN). Specifically, both targeting ligands were covalently tethered to the liposome exterior *via* DSPE-PEG2000 conjugation to form stable dual-targeted vesicles (Figure 5B). SC first binds to the ASBT highly expressed in the terminal ileum in the intestines to promote liposome penetration through the intestinal epithelium. Subsequently, these absorbed liposomes are transported to the liver *via* the portal vein and enter hepatocytes through binding to the Na^+^-taurocholate cotransporting polypeptide (NTCP) on the surface of hepatocytes to achieve targeted delivery. *Ex vivo* imaging confirmed that these surface modifications dramatically improved the structural stability and targeted tissue accumulation in the intestines compared to non-targeted liposomes (Figure 5C). Compared with unmodified and free NaA, the bioavailability of this liposome was increased by approximately 162% and 191%, respectively. Compared with free NaA, the serum half-life of this liposome was prolonged from 2.85 h to 15.58 h, the liver acetate accumulation was increased to 3.75 times that of free drugs, and the time to peak drug concentration in the liver was prolonged from 15 min to 60 min, significantly improving the retention and utilization efficiency of drugs in target organs (Hou et al., 2025). The multifunctional liposome platform constructed by Song et al. is a typical representative. This system adopts a triple synergistic design: first, using the polymer pHPMA to enhance the mucus penetration ability of liposomes; second, promoting cellular uptake through the R8 cell-penetrating peptide; finally, initiating efficient receptor-mediated endocytosis by means of the binding between deoxycholic acid (DOCA) and ASBT. To explicitly ensure surface presentation rather than membrane incorporation, DOCA was covalently anchored to the vesicle exterior using DSPE-PEG2000-DOCA conjugates. Compared with drug suspensions, the oral bioavailability of this freeze-dried liposome enteric-coated capsule was increased by approximately 151%, and the Tmax was prolonged by approximately 300%, fully verifying the key role of bile acids as explicitly tethered surface targeting ligands in complex multifunctional synergistic strategies (Song et al., 2025).

## 3 Challenges in clinical translation and industrial feasibility

Currently, while some simple oral liposomal formulations (such as nutritional supplements, vitamins, and liposomal iron) are commercially available and being evaluated in clinical settings, the clinical translation of advanced surface-modified liposomes for prescription drugs remains largely in the preclinical or early clinical trial stages (Davis et al., 2016; Bengelloun et al., 2024; Rangaraj et al., 2025). A notable exception is the recent progression into Phase I human clinical trials of novel oral lipid-based formulations (e.g., iCo-019 for the oral delivery of amphotericin B) (Hnik et al., 2020). Despite such encouraging milestones, bridging the gap between promising laboratory results and widespread clinical application requires overcoming several critical translational hurdles.

### 3.1 Industrial scale-up and manufacturing feasibility

Although the complex surface modification strategies discussed in this paper, such as LbL self-assembly and multi-ligand targeting, have demonstrated excellent barrier-overcoming capabilities in laboratory studies, their clinical translation still faces significant industrial production challenges. Traditional liposome preparation and multi-step coating processes often encounter bottlenecks when handling complex LbL systems, including difficulties in precise particle size control, susceptibility to vesicle aggregation, high polydispersity, and poor batch-to-batch reproducibility.

To address these scale-up challenges, microfluidics has emerged as a novel manufacturing approach exhibiting tremendous industrial potential. By precisely manipulating fluid flow rates and mixing ratios at the microscale, microfluidic technology enables the continuous preparation of highly monodisperse and size-tunable liposomes. For complex multi-layer coating and surface-functionalized systems, microfluidic platforms not only effectively overcome the limitations of traditional methods, but their continuous-flow production mode also supports seamless scale-up from the laboratory to large-scale manufacturing (Forbes et al., 2019; Lai et al., 2020).

Beyond particle assembly, the high cost of goods associated with synthesizing specific ligands (e.g., aptamers, antibodies) and the complexities of downstream processing remain significant economic barriers. These manufacturing complexities are fundamentally exacerbated by the inherent physical fragility of liposomes. Because their bilayers are assembled *via* non-covalent interactions, they are extremely sensitive to the intense mechanical shear stresses typical of large-scale industrial equipment, making them highly prone to structural deformation, vesicle disruption, and premature cargo leakage. Additionally, maintaining the structural integrity of multi-layer or ligand-decorated liposomes during long-term storage poses a further technical challenge for commercial viability. This final step frequently necessitates advanced lyophilization techniques along with the use of optimized cryoprotectants.

### 3.2 Safety and regulatory hurdles

The safety profiles of surface modification materials vary significantly. Natural biomacromolecules (e.g., chitosan, HA) and traditional polymers (e.g., PEG, Eudragit) generally have well-established safety records and long histories of biomedical use, making their regulatory approval relatively smooth.

In contrast, the clinical translation of novel synthetic polymers like PDA and PMO faces stringent biosafety and regulatory hurdles. For instance, despite its excellent mucus penetration, PDA may induce cytotoxicity or disrupt gut microbiota homeostasis and bile acid metabolism if unpolymerized monomers remain or high doses are used (Davidsen et al., 2021; Chen et al., 2023). Similarly, PMO relies on highly reactive groups, raising risks of off-target mucosal irritation (Qian et al., 2022). Because these novel excipients lack established pharmacopeial monographs, their *in vivo* degradation and long-term safety require comprehensive toxicological evaluations to meet FDA and EMA criteria. Additionally, upon repeated oral administration, the potential immunogenicity and degradation profiles of biologically derived ligands (e.g., proteins, antibodies) necessitate rigorous evaluation to prevent adverse immune responses.

To effectively overcome these translational barriers, future research must integrate advanced manufacturing technologies with rigorous regulatory-compliant frameworks. Specifically, coupling microfluidic platforms with Quality-by-Design (QbD) principles and Process Analytical Technology (PAT)—as strongly recommended by regulatory agencies like the FDA—can build a comprehensive understanding of the relationship between manufacturing conditions and final product characteristics. Implementing QbD and PAT facilitates the seamless scale-up of nanomanufacturing by ensuring real-time quality control and reproducibility (Agrahari and Agrahari, 2018).

Furthermore, to address the poor in vitro-in vivo correlation that frequently limits clinical success, it is crucial to transition from ordinary 2D cell cultures to advanced experimental models. Introducing 3D *in vitro* models (such as organoids and spheroids), vascularized co-culture systems, and patient-derived xenograft models can better mimic complex *in vivo* physiological conditions. These advanced preclinical models are essential for more accurately evaluating the biological interactions, safety, and efficacy of novel nanocarriers prior to human clinical trials (Younis et al., 2022).

## 4 Future perspectives: beyond multifunctional stacking

Moving beyond the practical hurdles of manufacturing and regulation, the next evolutionary step for oral liposomes lies in a fundamental shift in design philosophy. Reflecting on current advancements, we propose that the future trajectory of oral liposomes must pivot from mere “multifunctional stacking” to “spatiotemporally programmed” delivery capable of sequential barrier-crossing. Currently, simultaneously combining multiple ligands often leads to functional conflicts; for instance, strong mucoadhesion can inadvertently hinder the necessary mucus penetration required for deep epithelial access (an issue partially addressed by the adaptive systems discussed in Section 2.2.2.1). We believe the next major breakthrough lies in designing dynamic, sequentially responsive coatings. These intelligent systems should structurally evolve or shed specific layers strictly in response to the changing gastrointestinal microenvironment, utilizing precise triggers such as localized pH shifts, specific enzymes, or the unique metabolic activity of the gut microbiota.

Furthermore, from a translational perspective, a distinct tension remains between formulation complexity and clinical feasibility. While novel synthetic polymers like PMO or PDA offer remarkable laboratory efficacy, their rigorous biosafety and regulatory hurdles remain significant barriers that cannot be ignored. Therefore, we argue that future surface engineering should heavily prioritize the innovative utilization of endogenous or Generally Recognized as Safe (GRAS) biomaterials, including natural polysaccharides, dietary proteins, and bile acid derivatives. Focusing on these materials to achieve biomimetic targeting will effectively balance high delivery efficiency with superior clinical translatability, facilitating the next-generation of oral nanotherapeutics.

## 5 Conclusion

To overcome multiple challenges of oral liposomes, such as poor physicochemical stability and premature cargo degradation in the GI tract, mucus barrier obstruction, and low absorption efficiency, surface modification strategies have become core means to improve their delivery performance. The modification strategies comprehensively reviewed in this paper, as summarized in Table 2, mainly empower liposomes from three aspects: synthetic polymer modification (e.g., PEG, TPGS, Eudragit, PDA) mainly improves their physicochemical stability, cargo protective capabilities, and circulation time; biomacromolecule modification (e.g., polysaccharides, proteins, peptides, aptamers) focuses on enhancing mucoadhesion, cellular uptake, and active targeting capabilities; and small-molecule ligand modification (e.g., folic acid, vitamin B12, bile acid derivatives) achieves efficient absorption by utilizing endogenous transport systems. These strategies, either alone or in combination, have significantly improved the oral bioavailability of various model drugs.

**TABLE 2:** Summary of surface modification strategies for oral liposomes and their core functions.

Major modification strategy	Specific modification	Core function summary	Core mechanisms	References
Synthetic polymer modification	PEG	Enhances mucus penetration, stabilizes colloids, prolongs circulation time.	Forms a hydrated steric barrier; optimal penetration achieved with a transitional “mushroom-brush” conformation; reduces nonspecific interactions with mucus.	Singh et al. (2020b), Wu et al. (2022), Yamazoe et al. (2021)
​	TPGS	Inhibits P-gp efflux, enhances GI stability, promotes cell uptake and drug solubilization.	Acts as a P-gp inhibitor; forms a hydrophilic protective layer; improves drug release profile and intestinal cell internalization.	Wong et al. (2025), Wang et al. (2021a), Zhang et al. (2024)
​	Eudragit	Provides pH-responsive protection for gastric stability and site-specific (e.g., colonic) drug release.	Remains insoluble in gastric acid, dissolves at intestinal pH; enables targeted delivery and protects encapsulated cargo.	Nikam et al. (2023), Alizadeh et al. (2025), Pani et al. (2024)
​	Polydopamine (PDA)	Enhances mucus penetration and cellular uptake, improves drug-loading stability.	Exhibits superior mucus-penetrating ability compared to PEG; surface catechol groups promote cell interaction and internalization.	Poinard et al. (2019), Maurelli et al. (2024)
​	Poly (maleic anhydride-alt-1-octadecene) (PMO)	Achieves covalent bioadhesion to intestinal mucosa, prolonging local retention.	Maleic anhydride groups form covalent amide bonds with mucosal amines, significantly enhancing adhesion and residence time.	Qian et al. (2022)
Biomacromolecule modification	Chitosan and Its Derivatives	Enhances colloidal stability and mucoadhesion *via* electrostatic interaction; derivatives offer targeting or P-gp inhibition.	Cationic nature improves particle stability and mucoadhesion; thiolated derivatives inhibit P-gp; sugar-conjugated derivatives (e.g., mannose, galactose) enable receptor-mediated targeting.	Mohseni et al. (2025), El-Say et al. (2024), Arshad et al. (2024), Metkar et al. (2023), Fatouh et al. (2021)
​	Hyaluronic Acid (HA)	Improves colloidal stability and bioaccessibility; actively targets CD44 receptor-overexpressing cells.	Serves as a protective coating; binds specifically to CD44, enabling active targeting to inflammatory or cancer cells.	Sun et al. (2024), Chen et al. (2024), Wang et al. (2025)
​	Pectin	Forms a dense, protective coating, improving GI stability and enabling colon-targeting potential.	Electrostatic adsorption creates a stable layer; low-methoxyl pectins form particularly effective coatings; can be degraded by colonic microbiota.	Lutta et al. (2025), Su et al. (2024a)
​	Sodium Alginate (SA)	Enhances storage and GI stability, promotes sustained release and transmembrane penetration.	Forms a compact outer barrier, slowing drug release and protecting against enzymatic degradation.	Wu et al. (2023)
​	Double-Layer Polysaccharide	Significantly enhances GI tolerance, enables controlled colon-specific release *via* LbL assembly.	Multi-layer coatings (e.g., pectin/chitosan, chitosan/alginate) provide robust protection and pH/enzyme-responsive drug release in the colon.	Wang et al. (2023), Su et al. (2024b), Xian et al. (2021), Tan et al. (2023)
​	Proteins	Balances mucus penetration and cellular uptake; some possess enzymatic (mucolytic) or specific targeting functions.	Adaptive systems (e.g., protein corona) modulate surface properties for sequential barrier crossing; enzymes like bromelain digest mucus; specific proteins (e.g., SlpB) target immune cells.	Ding et al. (2024), Park et al. (2024), Tan et al. (2022)
​	Peptides (e.g., CPP, RGD)	Promotes cellular internalization/transcytosis; enables specific cellular targeting.	CPPs facilitate membrane translocation; homing peptides (e.g., RGD) target specific receptors (e.g., integrins on M cells).	Werner et al. (2024), Deng et al. (2024), Raut et al. (2023)
​	Aptamer	Provides high-specificity, high-affinity targeting to selected cell types (e.g., M cells).	Selected *via* SELEX to bind specific receptors, enabling precise active targeting and enhanced localized uptake.	He et al. (2023)
Small-molecule ligand modification	Organic Acids (e.g., Palmitic Acid)	Utilizes endogenous transport pathways (e.g., FATP4) to enhance uptake.	Serves as a substrate for nutrient transporters, potentially facilitating carrier-mediated transcellular transport.	Huang et al. (2025)
​	Folic Acid (FA)	Targets folate receptor-overexpressing cells (e.g., certain cancer cells, activated macrophages).	Enables receptor-mediated endocytosis, directing liposomes to specific cell populations for enhanced therapeutic delivery.	Yazdi et al. (2020), Li et al. (2023), Hong et al. (2025)
​	Vitamin B12	Targets the CD320 receptor for active absorption in the small intestine.	Exploits the physiological pathway for Vitamin B12 absorption *via* receptor-mediated endocytosis in the ileum.	Sarhadi et al. (2022)
​	Bile Acid Derivatives	Targets the ASBT for enhanced ileal absorption and liver delivery.	“Hijacks” the efficient ASBT-mediated transport pathway, facilitating liposomal entry into enterocytes and subsequent liver targeting.	Hou et al. (2025), Song et al. (2025)

Abbreviations: ASBT, apical sodium-dependent bile acid transporter; CPP, cell-penetrating peptide; FA, folic acid; LbL, layer-by-layer; PEG, polyethylene glycol; P-gp, P-glycoprotein; RGD, arginine-glycine-aspartic acid; SELEX, systematic evolution of ligands by exponential enrichment; TPGS, D-α-tocopheryl polyethylene glycol succinate.

In conclusion, surface modification engineering provides a powerful toolbox for precisely regulating the delivery behavior of liposomes, not only significantly improving the oral bioavailability of drugs but also laying a key foundation for their development into intelligent and targeted oral delivery platforms. Moving forward, while laboratory-scale outcomes are highly promising, successful clinical translation remains challenged by industrial manufacturing and regulatory hurdles. Future endeavors must integrate continuous scalable technologies, such as microfluidic platforms, with QbD principles and comprehensive biosafety profiling, which will ultimately bridge the gap between innovative formulations and clinically viable oral therapeutics. Most importantly, embedding these scalable manufacturing and safety considerations at the earliest conceptual stages of formulation design, rather than treating them as subsequent add-ons, will ultimately bridge the gap between innovative nanocarriers and clinically viable oral therapeutics.
